# The linguistic roots of natural pedagogy

**DOI:** 10.3389/fpsyg.2015.01424

**Published:** 2015-09-23

**Authors:** Otávio Mattos, Wolfram Hinzen

**Affiliations:** ^1^Grammar and Cognition Lab, Departament de Lingüística General, Universitat de BarcelonaBarcelona, Spain; ^2^Institució Catalana de Recerca i Estudis Avançats (ICREA)Barcelona, Spain; ^3^Department of Philosophy, University of DurhamDurham, UK

**Keywords:** language development, natural pedagogy, pointing, child communication, learning from communication, declarative gestures, concepts, knowledge about kinds

## Abstract

Natural pedagogy is a human-specific capacity that allows us to acquire cultural information from communication even before the emergence of the first words, encompassing three core elements: (i) a sensitivity to ostensive signals like eye contact that indicate to infants that they are being addressed through communication, (ii) a subsequent referential expectation (satisfied by the use of declarative gestures) and (iii) a biased interpretation of ostensive-referential communication as conveying relevant information about the referent’s kind (Csibra and Gergely, 2006, 2009, 2011). Remarkably, the link between natural pedagogy and another human-specific capacity, namely language, has rarely been investigated in detail. We here argue that children’s production and comprehension of declarative gestures around 10 months of age are in fact expressions of an evolving faculty of language. Through both declarative gestures and ostensive signals, infants can assign the roles of third, second, and first person, building the ‘deictic space’ that grounds both natural pedagogy and language use. Secondly, we argue that the emergence of two kinds of linguistic structures (i.e., proto-determiner phrases and proto-sentences) in the one-word period sheds light on the different kinds of information that children can acquire or convey at different stages of development (namely, generic knowledge about kinds and knowledge about particular events/actions/state of affairs, respectively). Furthermore, the development of nominal and temporal reference in speech allows children to cognize information in terms of spatial and temporal relations. In this way, natural pedagogy transpires as an inherent aspect of our faculty of language, rather than as an independent adaptation that pre-dates language in evolution or development (Csibra and Gergely, 2006). This hypothesis is further testable through predictions it makes on the different linguistic profiles of toddlers with developmental disorders.

## Introduction

In an article dedicated to explore some core similarities and differences between humans and non-human apes, Tomasello and Herrmann (2010) argue that our species have “more sophisticated cognitive skills for dealing with the social world in terms of intention-reading, social learning, and communication” (Tomasello and Herrmann, 2010, p. 5). The authors suggest that these skills are necessary for language but precede it in development (and presumably in evolution), as children can communicate before the emergence of speech through declarative gestures like pointing. In this way, they are already able to manifest to adults through pointing the referents about which they intend to communicate and learn. Language would add to this scenario other “fundamentally cooperative communicative devices – known as linguistic conventions (or symbols) – whose meanings derive from a kind of cooperative agreement that we will all use them in the same way” (Tomasello and Herrmann, 2010, p. 5).

The idea of a human-specific form of communication that precedes the emergence of language can also be observed in some archeologists’ interpretations of the archeological record of our hominin ancestors:

Could the (Neanderthal) knapper of Marjorie’s core have learned the significance and role of, say, the distal convexity without recourse to language? (…) We believe that the answer is yes. *If a teacher drew a novice’s attention repeatedly to the distal convexity (by pointing, for example), this would have been enough*. However, we believe that (this technology) would have been very difficult to learn without some sort of guided attention; it probably required active instruction, and *active instruction relies on joint attention and theory of mind. It does not require language*. (Wynn and Coolidge, 2010; our italics).

That said, we think that this thesis is *wrong* for reasons that we set out in this article. First, we will argue that the comprehension and production of declarative gestures by infants reflect structural aspects of human language. In particular, we suggest that declarative gestures are the first expression of determiner phrases in development, to which they are developmentally linked, corresponding to the assignment of the role of ‘third person’ in communicative acts. In combination with ostensive signals (like eye contact), which are used to define the initial first and second persons involved in communicative acts, declarative gestures in this way complete the ‘deictic space’ within which both natural pedagogy and language use naturally occur. Its foundations are centrally affected in infants with autism spectrum conditions, where not only the personal pronouns but also declarative gestures as well as determiner phrases at large can be affected (Lee et al., 1994; Modyanova, 2009; Hobson et al., 2010; Curtin and Vouloumanos, 2013; Shield and Meier, 2014; Hinzen and Schroeder, 2015).

Having linked the ‘pre-linguistic communication’ mediated by ostensive signals and declarative gestures to the faculty of language^1^, we will reflect on the kind of knowledge that children can acquire or convey through communication in light of the linguistic structures that emerge throughout the one word-period. We will suggest that at the ‘proto-determiner phrase stage’ children can only acquire knowledge that is generalized to kinds and that the emergence of the ‘proto-sentence stage’ in language development allows them to cognize information in terms of temporal and spatial relations — i.e., to “reconstruct from some parts of the adult’s (communication) a local, episodic content for the informative intention” (Csibra, 2010, p.157). However, children’s first assertions are bound to the here-and-now of speech. Language development not only expands these spatial and temporal limits, but also improves the capacity of children to understand and produce statements with sentential arguments that are anaphorically connected to entities and/or propositions that are given in the discourse.

We will argue for a faculty of language whose core function is to perform (through the production of linguistic structures) different referential acts in the spatial, temporal, and discourse domains, grounding all human-specific forms of referential communication — including infants’ use of declarative gestures. In this way, language would be inherent to human-specific aspects of communication from very early in development, instead of being a ‘tool’ designed by and at the disposal of human communication only at later ages. Our view contrasts with the perspective of formal linguistics, which has left the referential aspect of language largely aside during the last 50 years, confining itself to an ‘internalist’ inquiry as defined in Chomsky (2000). Independent linguistic evidence as synthesized in Hinzen and Sheehan (2013), however, suggests that the full spectrum of forms of reference available to humans patterns along with grammatical configurations, rather than being governed by non-linguistic factors. Reference is thus inherent to grammar.

This illustrates that we are not merely continuing the old Humboldtian debate about the relative primacy of either language or thought, by arguing in favor of a ‘language-first’ view. Instead we advocate that a specific capacity, namely natural pedagogy, is inherently integrated with language, making them two sides of the same human-specific coin. In this way there would be a single evolving system, and the prediction is that natural pedagogy and language will never dissociate. An obvious way to explore this hypothesis further empirically is to compare typically developing children and children with communicative disorders regarding their capacity to learn different kinds of information through communication. In such a study, we would expect that particular problems in language development (e.g., a delay in the individual onset of proto-determiner phrases and proto-sentences) would be significantly associated to an atypical development of natural pedagogy (see Language and Learning from Communication as Two Non-Dissociable Capacities).

Connecting language to natural pedagogy could also motivate a new proposal within the currently stagnant debate about the origins and evolution of our linguistic capacity (Hauser et al., 2014). In contrast with living non-human apes who basically learn traditions *emulating* older generations — i.e., trying to reproduce the end result of actions through trial and error method (Tennie et al., 2009)^2^ — communication is the main source of knowledge for humans (Coady, 1973). If linguistic structures are *inherent* to human-specific forms of communication as we here defend, then in exploring these structures we could understand better the main “social-cognitive skills that enable (humans) to develop, in concert with others in their cultural groups, creative ways of coping with whatever challenges may arise” and “deal with everything from the Arctic to the tropics” (Tomasello and Herrmann, 2010, p. 7). Perhaps the emergence of the so-called Mousterian stone tool technology in hominin evolution relied on this human-specific mechanism — after all, it succeeded the Acheulean technology, which is the stone tool tradition that has remained the longest in human evolution and yet “true and persistent innovation does appear to be lacking” in it (Ambrose, 2001; Nowell and White, 2010, p. 76). If we can show that the faculty of language is not simply ‘a symbolic system’ (an idea that perhaps is implicit in Csibra and Gergely, 2006, and in Tomasello and Herrmann, 2010) but *the* symbolic *and* referential system behind all human-specific forms of referential communication, the interpretation given by Wynn and Coolidge (2010; see above) that pointing “would have been enough” to teach apprentices how to produce the Mousterian tool in question would favor our hypothesis that at least a proto-language was in place by that point.

In summary, we will argue here for a faculty of language as a ‘non-encapsulated’ universal capacity that is inherent to aspects of communication and meaning that are human-specific — and we will do so by focusing on a core capacity for humans, namely natural pedagogy. In order to ground the present perspective, in the first section we will explore the connections between declarative gestures and the faculty of language in more detail, while in the second our focus will be on the relation between different linguistic structures and the kinds of knowledge that children can acquire or convey through communication. We will conclude by suggesting that human communication — and specifically our species-specific capacity to acquire cultural knowledge through it — is deeply rooted in the faculty of language.

### Declarative Gestures: Language’s Illegitimate Child

Csibra and Gergely (2006, 2009, 2011) state that only humans among all living species have *natural pedagogy*: i.e., the capacity to transmit cultural knowledge through communication to new generations and the capacity of new generations to learn cultural knowledge from communication. Briefly, an adult manifests his communicative intention to a child by directing an ‘ostensive signal’ (e.g., eye contact) to her and then the child instinctively expects to receive new information about some object in the immediate surrounding world — a piece of information that she generalizes to every object of the same kind. Evidence shows that by 4 months of age infants already react to adult ostensive signals, but only by 10 months of age do these stimuli induce them (i) to expect and follow declarative gestures like pointing or gaze-shift to identify a referent in the world and (ii) to consider the adult’s attitude toward the referent an informative behavior (Csibra, 2010). In other words, at 10 months of age infants expect and come to be part of a ‘deictic space’ within which cultural information can be acquired by connecting the third person (established at this moment exclusively through declarative gestures), the second and the first person (established through ostensive signals).

Csibra and Gergely (2006) argue that declarative gestures are our earliest form of referent assignment not only in development, but also in evolution. These gestures and broadly speaking “the ability to teach and to learn from teaching (are) a primary, independent, and possibly phylogenetically even earlier adaptation than language” (Csibra and Gergely, 2006, p. 2). Within this view, only symbolic and iconic gestures, but not indexical gestures like pointing, would be associated to language. Our goal in this section is to challenge this statement, presenting evidence that the human use of indexical gestures and natural pedagogy reflect structural aspects of language.

The relation between declarative gestures and language development has been explored in many studies (see for example Butterworth and Morissette, 1996; Markus et al., 2000). Colonnesi et al. (2010) examined twenty-five of these studies (734 children in total), concluding that pointing is related to speech both longitudinally and concurrently: (i) longitudinally, the amount of pointing produced by infants predicts their speech production rates (see also Butterworth, 2003) and (ii) concurrently, pointing is used in integration with speech. Importantly, they found statistically significant associations between declarative pointing and language already by 10–11 months of age — when infants start to produce declarative gestures but still do not produce words — and the strongest associations between 15 and 20 months of age. These associations were found for declarative pointing (i.e., a gesture that ‘declares’ a referent, e.g., when a child points at a dog) but not for imperative gestures (i.e., a gesture that children use to induce others to take an object for them, using other people as tools to solve an immediate problem).

Children first start to produce co-speech gesture combinations to convey ‘reinforced information’ — for example, pointing at a dog and saying ‘dog’ — and only later in development they produce ‘supplemented information’ — for example, pointing at a dog and saying ‘go,’ a kind of combination in which each modality (speech and gesture) conveys different pieces of information (Goldin-Meadow and Butcher, 2003; Iverson and Goldin-Meadow, 2005; Özçalɪşkan and Goldin-Meadow, 2009; Cartmill et al., 2014). Importantly, the emergence of the latter never precedes the former in development, and each of these combinations predicts the individual onset of specific linguistic structures in speech, i.e.: the individual onset of reinforced co-speech-gestures predicts the individual onset of determiner phrases in speech, while supplemented co-speech-gestures predict the individual onset of sentences in speech (Goldin-Meadow and Butcher, 2003; Özçalɪşkan and Goldin-Meadow, 2009; Cartmill et al., 2014). The successive emergence of ‘proto-determiner phrases’ and ‘proto-sentences’ in the one-word period moreover parallels the fact that the words that children are producing at around 14 months of age are nouns related to people (e.g., ‘baby,’ ‘dad’ etc.), objects (e.g., ‘banana’) and animals (e.g., ‘rabbit’), and expressive utterances like ‘hello,’ while only at around 19 months of age do they start to produce verb-like words like ‘woof’ and ‘yes/no’ answers — a developmental pattern observed in signing and speaking children alike, as well as in monolinguals and bilinguals (Nelson, 1973; Holowka et al., 2002)^3^.

When humans produce or comprehend declarative gestures they are necessarily connecting referents in the external world to concepts in their internal world. Natural pedagogy can only transmit knowledge about kinds because such a connection exists. Our claim here is that the mechanism underlying this bridge between external and internal world *is* the faculty of language, which is our symbolic and referential system *par excellence*: the development of this very faculty leads children from the use of declarative gestures — alone or combined with meaningless vocalizations or one-word utterances — to a more complex set of ‘resources’, by which different forms of reference (such as nominal and temporal reference) and concepts can be linked in multiple ways, giving rise to a pedagogy that conveys different kinds of information^4^. This is why declarative pointing and speech are strongly related along development^5^, and, as we will suggest in the remainder of this section, this is also the reason why non-human animals (chimpanzees, cats, dogs, dolphins etc.) *do not* produce or comprehend declarative gestures (pointing and gaze following) in the same way that infants at 10 months of age do.

Evidence demonstrates that chimpanzees do not comprehend pointing as a declarative gesture (Povinelli et al., 2003; Miklósi and Soproni, 2006). Povinelli et al. (1997) trained seven chimpanzees to use experimenter’s pointing gestures to locate a treat hidden in one of several possible locations. After many trials, the apes responded to these gestures very accurately, so the researchers increased the distance between the correct location of the treat and the distal end of the experimenter’s pointing. In this situation, the success rate of five of the seven chimpanzees decreased from 100% of correct choices to chance levels, making the researchers conclude that “apes were simply focusing on the local configuration of the experimenter’s hand and the box” (Povinelli et al., 2003, p. 60). However, since two apes still performed above the chance level, the researchers conducted a new experiment with the seven chimpanzees: in one case the experimenter was closer to the incorrect location and in another case the tip of the experimenter’s finger was equidistant from the two possible locations (in both cases, of course, the experimenter was pointing to the correct location). Results showed that all chimpanzees made the wrong choice in the condition where the experimenter was placed closer to the incorrect location; in the other condition, all apes performed randomly. Finally and essential to our discussion, the authors also observed that 3 year-old children were perfectly accurate from the first trial onward in the same experimental procedure^6^.

The study of Povinelli et al. (1997) thus shows that after much training chimpanzees can learn that some perceptual aspects of the experimenter’s physical disposition can be used as ‘hints’ to determine the location of the treat — strongly contrasting with infants, who spontaneously start comprehending and producing declarative gestures by 10 months of age (Butterworth, 2003; Cartmill et al., 2012). On the other hand, chimpanzees seem to perform much better in tasks involving gaze and head movement: they follow experimenter’s line of sight even when it projects outside their perceptual field (an ability that emerges in children only by 18 months of age; Butterworth, 2003) and they also take into account that this line of sight can not cross opaque screens (Povinelli and Eddy, 1996). Can this be evidence that chimpanzees comprehend other’s gazing at a target as a declarative gesture, just as humans do?

We believe that the answer is no, but before explaining our position we also want to consider briefly the ability of some non-primates to take into account human pointing gestures. Cats, dogs, dolphins, and seals perform the experiment described before (Povinelli et al., 1997) much better than chimpanzees — and they do it at a high level from the beginning of the test, just like children (Miklósi and Soproni, 2006). Furthermore, dogs seem to improve their performance even more when the pointing gesture is preceded by eye contact (Miklósi and Soproni, 2006) — which is a strong parallel with children’s sensitivity to adults’ ostensive signals. All this raises the question whether both sensitivity to ostensive signals and declarative gestures, far from being specific to humans, might be something that can independently emerge in cooperative species (e.g., dolphins) and/or can be the evolutionary consequence of domestication (which would also explain that dogs realize better than wolves the mentioned experiment) (Miklósi and Soproni, 2006; Topál et al., 2009).

The main problem for this line of thought is that these experiments do not show that the same interpretative bias lies behind the correct behavioral response of chimpanzees, dogs, and infants (Povinelli and Eddy, 1996; Topál et al., 2009). For example, babies at 6 months of age also seem to be able to follow adults’ gaze (Butterworth, 2003), but they do this differently from infants at 10 months of age, in two respects: firstly, the precise identification of the target is determined by the salience of the object in the situation — a mechanism that Butterworth (2003) called ‘ecological mechanism of joint visual attention.’ In our view, an analogous ‘ecological mechanism’ can be suggested for animals like dogs: they seem to try to satisfy instructor’s expectation taking to him (or finding) some salient object whose location is indicated by pointing or gazing (Topál et al., 2009). Secondly, we use declarative gestures for more than directing others’ attention to salient objects, and infants by 10 months of age are aware of this: they expect to receive new information about the kind of the assigned referent.

Therefore, both chimpanzees and dogs are able to perform as well as infants in tasks involving, respectively, gaze following and pointing, and both seem to be sensitive to ostensive signals (Miklósi and Soproni, 2006), but we have seen that only in the case of the infants, ostensive signals make them expect the transmission of new information about the kind of the assigned referent. This can be explained in light of the faculty of language, which is at the same time a referential *and* a symbolic system — i.e., a system that connects the external world to our internal, conceptual world. Although infants by 10 months of age still do not produce words, this system has already started to develop: they can only acquire knowledge about *kinds* because (i) they hold concepts in relation to these kinds, and (ii) they can link these concepts to assigned referents in the situational context (Hinzen and Sheehan, 2013:ch. 2; Bickerton, 2014).

The use of artificial language by apes illustrates very well the unique character of human language as a referential and conceptual system. Cartmill and Maestripieri (2012) observed that apes can use arbitrary gestural symbols that are not linked to internal states like emotions, they can map these symbols to objects of the world and they can learn these symbols from passive observation. However, the authors affirm that although apes are (i) “provided with individual units that are analogous to human words (i.e., referential, arbitrary, taught)” (Cartmill and Maestripieri, 2012, p. 19), they (ii) “do not display any aptitude in combining the units in a systematic or meaningful way.” The problem here is that reference emerges in human language only *from* the structure of phrases, not from words alone (Rozendaal and Baker, 2008, 2010; Martin and Hinzen, 2014), therefore being able to “combine the units in a systematic or meaningful way” (ii) is a necessary condition for human referentiality. For example, the arguments of the sentences ‘a cat meows,’ ‘the cat meows’ or ‘this cat meows’ are not ‘referential isolated words’ but determiner phrases — i.e., they combine referential operators with nouns. In short, the word ‘cat’ alone is not referential at all. Furthermore, the position of the determiner phrase in the sentence structure can prevent its referentiality — e.g., in ‘a thief entered,’ the determiner phrase ‘a thief’ picks out a referent, while in ‘that guy is a thief’ the same determiner phrase ‘a thief’ works as a predicate (picking out a property ascribed to a referent instead of a referent).

Referentiality in humans is a combinatorial phenomenon *par excellence*, therefore an inaptitude in “combining the units” suggests that apes cannot display the kind of referentiality produced by human language either. This combinatorial aspect of human referentiality explicitly guides infants’ use of declarative gestures: at the beginning these are often produced with meaningless vocalizations (Cartmill et al., 2012), which gives place to one-word utterances by 12 months of age — importantly, children’s initial vocabulary seems to be related to the number of different kinds of objects that they point to before the one-word period (Iverson and Goldin-Meadow, 2005), which indicates that lexical concepts are already in place at this moment, being combined with declarative gestures in children’s communication. In the terms of Martin and Hinzen (2014), in a definite description like ‘the dog,’ the determiner ‘the’ is the ‘edge’ of the phrase and regulates its referentiality (determining definiteness in this instance), while ‘dog’ is the ‘interior’ of the phrase and determines the descriptive content involved in the act of reference. Therefore, infants’ declarative gestures express the referential *edge* of the determiner phrase, while their words (pronounced or not) are related to the conceptual *interior* of this nominal structure (which is linked to their knowledge about kinds)^7^. In short, while Cartmill and Maestripieri (2012) state that non-human apes can use an artificial language referentially but not combinatorially, we state that human language is referential because it is combinatorial — not combinatoriality in a generic sense (of a type, for example, that can be found in artificial languages or music as well), but related specifically to grammar, which correlates with the genesis of referentiality in language.

To stress our point, we agree with Petitto (2000, p. 383) that it remains uncontroversial that “all chimpanzees fail to master key aspects of human language structure, even when you give them a way to bypass their inability to speak — for example, by exposing them to (…) natural signed languages” (see also Tomasello, 2008). For her, and for us as well, this indicates that chimpanzees lack cross-modal mechanisms that ground the development of both signing and speaking of any natural language, rather than merely mechanisms for perceiving and expressing speech sounds. In our view, however, these cross-modal linguistic mechanisms do not only involve the necessary ability to “detect aspects of the patterning of language (…) the temporal and distributional regularities initially corresponding to the syllabic and prosodic levels of natural language organization” (Petitto, 2000, p. 397), but also the capacity to perform reference — indeed, this referential mechanism seems to play an important role in the acquisition of native phonetic structures: at 9 months of age, infants enhance the discrimination of sounds that co-occur with distinct referents (Yeung and Werker, 2009), at the same time that their ability to statistically learn phonetic categories starts to decrease (Yoshida et al., 2010).

The combinatorial nature of referentiality in humans (i.e., a referentiality grounded on linguistic structures formed by a referential edge and a semantic interior) explains a further, long-noted aspect of ‘intentionality’ (with a ‘t’), namely ‘intensionality’ (with an ‘s’), which is induced by the lexical description of the nominal phrase. By (human) intentionality (with a ‘t’) we mean the deliberate reference to things based on internal concepts, while intensionality (with an ‘s’) arises because, if I know a referent under one description, I may of course not know it under an indefinite number of others — in other words, descriptions applicable to the same referent could be non-equivalent in the subject’s mind. Thus I may not know that a colleague, Mr. Smith, is also my wife’s secret lover, or my daughter’s most hated teacher. My thought or statement that Mr. Smith is an honorable gentleman is therefore inaccurately (or at least misleadingly) reported as the thought or statement that my wife’s secret lover is an honorable gentleman, even if the two descriptions pick out exactly the same man. Now, it would be equally misleading for someone to say, if I *point* to what is (for me) Mr. Smith, that I pointed to my wife’s lover: the description stands between the referent and the person referring, as it were, and also in pointing, reference is systematically dependent on description. If declarative gestures exhibit intensionality in this sense (and consequently intentionality, as the latter is inherent to the former), it is hard to see how they are not inherently linguistic, given the inherent difficulty of establishing intensionality for any non-linguistic animal (Davidson, 1982)^8^^,^^9^ .

Natural pedagogy, then, could, as we have argued, be the comprehension side of a coin that has proto-determiner phrases as its production side. Through natural pedagogy, infants connect assigned referents in the external world to concepts in the internal world, promoting an ‘exchange’ in which their current knowledge ‘explains’ the stimuli and interlocutors’ behavior toward the stimuli modifies infants’ current knowledge. The emergence of proto-sentences in language development will be equally related to the emergence of a new pedagogy: one that is based, as we will argue in the next section, on the transmission of knowledge about *facts*.

Therefore, if we take as ‘declarative’ only the gestures that are used as expressions of nominal ‘edges,’ linking the external world to our conceptual/internal world, these gestures are not only human-specific but linguistically based. In this way we disagree with views that describe declarative gestures as merely something used to “re-direct(s) the partner’s attention to some distant object or event” (Leavens, 2004, p. 395). This is a necessary but not a sufficient condition for declarative gestures in the sense that we have assumed here. ‘Declarative gestures’ as defined by Leavens (2004) can be comprehended by distantly related species like dogs, cats, dolphins, seals, and also chimpanzees (in this latter case only gaze and head movement), hence a necessary distinction is missed. Declarative gestures in our sense seem to have only emerged in hominin evolution, being not only related to the emergence of natural pedagogy but also to the emergence of a (proto-) language that allowed our ancestors to produce (at least) proto-determiner phrases^10^.

In the following section we will try to demonstrate that natural pedagogy can be better understood if we take into consideration the specific developmental stage of language that parallels its emergence. In doing so, we will be able to not only understand natural pedagogy but also the emergence of other forms of communicative learning.

### Language and Learning from Communication as Two Non-Dissociable Capacities

In this section we will defend the hypothesis that the faculty of language and the capacity to learn from communication are intrinsically related. In order to do so, we will argue that the earliest form of communicative learning to emerge in development — natural pedagogy — can be better understood in light of the first kind of linguistic structure that infants produce — namely what we called proto-determiner phrases. On the other hand, the emergence of sentence-like structures in language development gives rise to another form of ‘pedagogy’: one that conveys information about particular events, actions, and state of affairs. Both pedagogies presuppose a ‘communicative triangulation’ between the speaker (the grammatical first person), a hearer (the second), and an assigned referent (the third), but only sentential structures can produce statements about the world, statements that, by their very nature, can be true or false. Finally, we will show that language development gradually frees children’s statements from their temporal, spatial, and anaphoric ties, allowing them to talk about entities that are not physically present in the situational context, events that happened or will happen in a remote past or future and entities and/or claims that were previously mentioned in a conversation.

Csibra and Gergely (2006, 2009, 2011) point out that natural pedagogy is specific to humans, not because no animal can communicate or learn, but because they are not able to learn generic knowledge *from* communication. The problem is that animal forms of communication like alarm calls (i) always convey fixed configurations of message and referent and (ii) are always restricted to the immediate situation of subjects — for example, they alert conspecifics to the presence of predators, indicating with a single signal that, say, an aerial predator is approaching (Csibra and Gergely, 2011). Natural pedagogy, however, can convey a potentially infinite set of information about the same referent, and this information is generalized to other objects of the same kind. In other words, we can point at a bird and communicate many different things about it, and the hearer will consider this information in other moments and places for the same kind of entities. This suggests that at the proto-DP stage, where sentential configurations are still missing, new information is not actually tied to time and space. As we shall see below, what changes in the proto-S stage are not the elements of abstraction (e.g., lexical concepts) — they entail, *ipso facto*, generality, and function predicatively even in the proto-DP stage —, but children’s capacity to grammatically cognize temporal and spatial relations through sentences.

As noted, humans use ostensive signals (e.g., eye-contact) to demonstrate their communicative intention to an interlocutor (Csibra, 2010), and adult ostensive signals cause infants from approximately 10 months onward not only to follow their deictic gestures (like gaze-shift or pointing) but to expect novel information about the referent’s kind. Furthermore, infants within ostensive communication assume that this novel information is available for everyone — reacting when subjects other than the interlocutor do not take the generic information into account (Gergely et al., 2007). In this way, infants do not relate interlocutors’ positive attitude toward, say, a plate of broccoli to his or her mental state, but to the properties of broccoli as a kind (e.g., ‘broccoli is good’), and consider that this property is available to other subjects as well.

Our hypothesis is that children’s capacity to acquire and transmit knowledge through communication develops in connection with language. In this way, natural pedagogy is related to the emergence of proto-determiner phrases and this very fact gives us insight into why natural pedagogy transmits generic knowledge about kinds. The explanation is the following: sentence structures, but not determiner phrases, relate information to sentential arguments and to a time span — i.e., a time that can precede, contain or follow the time of utterance, as in the past-tensed statement ‘the book was on the table’ (Klein, 1998, 2006). Therefore, when acquiring knowledge through natural pedagogy, infants seem to take assigned referents as ‘physical expressions of concepts,’ in such a way that any new information about these referents automatically constitutes new information about the concepts to which these referents are associated. The needed sentential complexity to restrict a predicate to a time and context is simply not yet there.

Relating natural pedagogy to the proto-DP stage can also explain why 12 months old infants seem to point declaratively essentially to obtain generalizable information about the world and not to inform interlocutors about the situational context (Southgate et al., 2007). In our view, children can only inform others when they are able to take referents as arguments of sentential predicates — as in the case described by Lock (1997) in which a child uttered the word ‘dog’ and, when her mother asked ‘what is the dog doing?’, she said ‘woof’. Before that, however, they use declarative gestures exclusively to indicate the objects of their interest, stimulating adults to convey new information about their kinds. This is indeed the only scenario that we could expect. If children at the proto-DP stage can only extract generic knowledge from communication, how could they convey non-generic information about the situational context?

For this reason, we think that we should nuance Csibra’s and Gergely’s (2006, p. 6) argument that natural pedagogy is connected to “the predicate-argument (knowledge-referent) structure of human communication.” This is true if we consider that natural pedagogy involves the connection of properties (semantic/conceptual knowledge) to referents, but false if we imply from this that semantic content and referents are connected through *sentence-like* constructions as this kind of structure only emerges in child development by 18 months of age (i.e., approximately 8 months after the emergence of natural pedagogy) (Goldin-Meadow and Butcher, 2003; Iverson and Goldin-Meadow, 2005; Özçalɪşkan and Goldin-Meadow, 2009). Suggesting that natural pedagogy involves *sentential* predicate-argument structures would go against the developmental pattern of language described in the previous section and undermine a linguistic explanation for the human-specific capacity to acquire, through communication, different kinds of information — respectively, *knowledge about kinds* and knowledge about particular events, actions and state of affairs, which we will call here simply *‘knowledge about facts.’* From this perspective we hypothesize here that at the DP-stage children would be able to learn through communication that ‘broccoli’ (as a kind) is good but not that something specific happened to her plate of broccoli, like that it fell down. The onset of the latter capacity would predict (or would be predicted) by the onset of proto-sentence production.

We currently explore this hypothesis through a longitudinal study that aims to (i) analyze children’s production of gestural and oral communication throughout the one-word period and (ii) verify children’s capacity to acquire information about specific events, using a version of Ganea et al.’s (2007) experimental design with stuffed toys. In their study, infants were told that a particular stuffed toy that had been earlier named had undergone a change in state while out of view. Subsequently, the infants’ capacity to identify it exclusively on the basis of its new state was verified. Although the aim of the authors was to check children’s capacity to incorporate “(communicative) information into one’s mental representation of the absent object,” we have decided to go one step further and see if children’s success in this test is significantly correlated to the individual onset of proto-sentence production. We also involve children with communicative disorders, specifically regarding their production of communicative gestures (i.e., declarative, descriptive, and symbolic gestures) and words.

An essential distinction between knowledge about kinds and knowledge about facts is that only the latter could bear truth value: it is connected to sentence structures, which is our only means to acquire and convey true/false information about the world^11^. This seems to be in consonance with Prasada (2000, p. 67), who says that a key aspect of knowledge about kinds is that “(it is) not rendered false by the existence of instances that lack the essential property” (e.g., the existence of a three-legged dog does not make us to abandon the idea of dogs being four legged^12^). In this way, the production of sentential structures by the human mind would not be necessary for the acquisition of generic knowledge about kinds through communication, although, of course, we can express generic information through them (e.g., ‘dogs are four-legged’).

Determiner phrases allow us to cognize object reference but not temporal reference^13^ — which is a fundamental component of non-generic statements (Klein, 1998, 2006; Sheehan and Hinzen, 2011; Martin and Hinzen, 2014). When adults make claims about particular events or situations, these are always referred to as preceding, containing or following the time of utterance (Bonomi, 1995; Klein, 1998, 2006), in such a way that the truth of these assertions are limited to their specific ‘temporal frames’. For example, if I say ‘Cristina was drunk,’ the finite verb ‘was’ indicates that this claim is about a situation that *precedes* the time of utterance, therefore shifting temporal reference to the past and restricting truth to this time span. Importantly, that ‘Cristina *was* drunk’ is true does not indicate that ‘Cristina *is* drunk’ is necessarily false: ‘was’ does not establish when the situation ends, it only indicates *for which time span the state of affairs described by the statement is supposed to be assessed as true*^14^.

Someone could suggest that the so-called ‘tenseless languages’ challenge our hypothesis about the intrinsic connection between assertion and temporal reference in grammar. Speakers of, for example, Germanic and Romance languages use finite morphology to produce the time span of events referred to in assertions, but languages like Yucatec Maya (Bohnemeyer, 2009) and Tupí-Guarani (Tonhauser, 2011) are said to be tenseless. However, the question in these cases is how interlocutors connect statements to time spans and not whether these statements are or are not linked to them (Bohnemeyer, 2009). In this way, for our purpose it is enough to say that languages have different forms to encode the time span of assertions and that these forms emerge gradually in language development.

Another possible criticism is that linguistic resources like finite morphology and temporal adverbs do not emerge when children start to make assertions either (Blom, 2003; Dimroth et al., 2003; Jolink, 2005), and therefore their claims would not be circumscribed to any temporal frame. Evidence nevertheless shows that children’s untensed claims are by default related to the time of utterance: from the proto-sentence stage to approximately 31 months of age, children seem to only make claims about events, actions, and state of affairs that happen at around the moment of their speech (Morford and Goldin-Meadow, 1997). The ability to make reference to remote events in the past or future seems to be related to the development of finiteness in language, which starts to emerge by 24 months of age and is fully mastered by 36 months of age (Blom, 2003; Dimroth et al., 2003; Jolink, 2005).

Morford and Goldin-Meadow (1997) also noted that the home-signing deaf children in their study, despite the lack of a conventional language model to learn from, first started to talk about events that happened or were about to happen at around the time of their Signing and only later did they communicate about events in a distant past or future. Therefore, although the lack of linguistic input seems to have delayed the maturation and performance of temporal reference in the home-signing deaf children of the study — they talked about both near and distant events less often, and started to do it over a year later compared to hearing children —, the development of temporal reference followed the same stages observed for hearing children. It therefore appears that temporal reference is such a fundamental milestone in the development of the faculty of language (and consequently, of human communication) that even in the absence of linguistic input, the home-signing deaf children developed their own means to talk about remote past or future events — e.g., creating novel gestures, adapting some conventional gestures from their hearing community in order to mark temporal displacement.

Apart from releasing children’s statements from their ‘temporal ties,’ language development also frees them from their ‘spatial’ and ‘anaphoric’ constraints. Let us consider the following example: ‘A racoon chased the cat.’ In this sentence, the indefinite noun phrase “a racoon” introduces a new referent into the conversation — in languages like English and French, indefinite noun phrases cannot be used to refer to given referents (De Cat, 2004; Rozendaal and Baker, 2008) —, while the definite noun phrase ‘the cat’ either refers to a given referent in the discourse (i.e., to a cat that was previously mentioned in the conversation) or to a cat that the interlocutors mutually know from before (Rozendaal and Baker, 2008). In relation to adding new referents to a conversation, we have seen that children at the one-word period still do not use indefinite or definite noun phrases to assign referents but rather use declarative gestures, which makes these toddlers highly dependent on the situational context^15^. With regards to anaphoric reference to elements (entities or propositions) that were previously mentioned in a conversation, children simply seem to omit them in their utterances (as in the example mentioned before in which the child said just ‘woof,’ omitting the agent of the action (the dog) that was already referred to in her conversation with her mother). This represents an insuperable barrier for managing conversations with many competing given referents, as probably is the case of most adult conversations — indeed, this seems to be a problem even for children at the beginning of the multi-word period (Salazar Orvig et al., 2010).

In this way, at the beginning children’s statements are completely related to the here-and-now of speech and generally restricted to few (if not a single) referent. Then, throughout language development, children gradually shed these ties. By 24 months of age they start assigning referents that are not necessarily present in the situational context through determiner phrases in speech, and by 31 months of age they start to talk about events located in a remote past or future through linguistic resources like tense morphology, temporal adverbs etc. Finally, the emergence of anaphoric resources in language allows children to grammatically articulate different given elements of a conversation in new, asserted information — as in the case of the simple sentence ‘she did it’ (Lambrecht, 1994) in which all constituents have an anaphoric form but the sentence itself adds a new fact for the interlocutor.

To summarize, we have argued in this section that knowledge about kinds is grounded on (proto-)DP structures, which emerges approximately 8 months before (proto-)sentences in development. Only sentence structures can bind information to a time span and to sentential arguments, and this is the reason why the knowledge conveyed through natural pedagogy is never restricted to the referent in the situational context but generalized to all other objects of the same kind. Furthermore, we also argued that the development of linguistic resources for nominal and temporal reference in speech not only frees child statements from their spatial and temporal ties, but also allows children to grammatically connect their assertions to entities and/or propositions that were previously mentioned in a conversation. All in all, therefore, language and communicative learning go hand-in-hand in a very specific sense: the kind of knowledge that humans can exchange through communication is grounded on the linguistic structures that we are able to cognize in the course of development. In our view, communicative learning is rooted in the faculty of language rather than being a different and unconnected human-specific trait. This is a parsimonious conclusion considering that, in general, evolution is a conservative process, which means that “novel applications generally arise via utilization of preexisting mechanisms” instead of “depending upon *de novo* mutation and selection” (Richman and Naftolin, 2006, p. 7).

## Conclusion

We have defended a perspective in which language and learning from communication form two non-dissociable capacities. From this perspective, natural pedagogy represents an initial challenge, since it was originally proposed as a non-linguistic (although human-specific) capacity, both in development and evolution (Csibra and Gergely, 2006). However, we have argued in Section “Declarative Gestures: Language’s Illegitimate Child” that declarative gestures — fundamental for natural pedagogy as they are the first form of referent assignment that infants can understand and produce — are the Achilles heel of this hypothesis. Firstly, children’s initial vocabulary seems to be linked to the number of different kinds of objects that they point to before the onset of the one-word period (Iverson and Goldin-Meadow, 2005), which indicates that lexical concepts are being combined with declarative gestures at this moment. Furthermore, although by 10 months of age infants are still unable to produce words, they have started to understand lexical concepts insofar as they acquire generic information about referents’ kinds. These symbols are also behind both, the intentionality (with a ‘t’) and intensionality (with an ‘s’) of declarative gestures. We have seen in Section “Declarative Gestures: Language’s Illegitimate Child” that, despite the fact that animals like dogs seem to be sensitive to ostensive signals and to understand the directionality of pointing, they never expect to receive new, generic information from communication (Miklósi and Soproni, 2006; Topál et al., 2009). Humans seem to comprehend declarative gestures in a way that can only be explained in light of a system that is symbolic and referential at the same time, a system that no other living animal has. Evidence and parsimony suggest that language is the best candidate that we can appeal to in this regard.

Moreover, combinations of declarative gestures and lexical concepts obey a developmental pattern: children start combining pointing and isolated words to ‘reinforce’ the identity of referents in the situational context — e.g., pointing at a dog plus the word ‘dog’ — and only later in development do they combine gesture and isolated words to produce ‘supplementary’ meaning — e.g., pointing at a dog plus the word ‘go’. We’ve seen that the individual onset of these stages predicts, respectively, the individual onset of determiner phrases and sentences in two-word speech, the reason why we called them proto-DP and proto-S stages.

In the same way that natural pedagogy and the proto-DP stage are two sides of the same coin, the emergence of the proto-S stage in development gives rise to a pedagogy with new properties. While natural pedagogy conveys *knowledge about kinds*, the pedagogy based on sentence structures conveys *knowledge about facts*. Knowledge about kinds would be not only generic but unfalsifiable, while knowledge about facts can be non-generic and falsifiable — being bound both to sentential arguments (expressed through definite and indefinite noun phrases, bare plurals, pronouns etc.) and to verbal inflections that specify for which time span the piece of information is supposed to be assessed as true (the past, present, or future of the time of utterance). For example, from our perspective children’s capacity to understand through communication that a specific stuffed toy has fallen or got wet would rely on their mental ability to build sentence structures — a prediction testable in different populations, as noted.

Furthermore, we tried to explore in more detail the proto-DP and proto-S stages that we outlined in Section “Language and Learning from Communication as Two Non-Dissociable Capacities”. First, we have seen that at the proto-DP stage, infants and young children are able to introduce referents for a conversation, but they cannot talk about them. The reason for us is related to the fact that they still do not produce sentential predicate-argument structures. Second, we have argued that at the beginning of the proto-S stage, children’s statements are bound to the place and moment of the conversation: they can only introduce referents through declarative gestures and their statements are never related to a remote past or future (Morford and Goldin-Meadow, 1997). The more the use of determiner phrases and finiteness in speech increases, the more communication becomes *relational* —allowing children to introduce referents that are not present in the situational context (i.e., the ‘here’ of the interlocutors) and to talk about distant events in the past or in the future (i.e., the ‘now’ of the interlocutors) (Morford and Goldin-Meadow, 1997; Rozendaal and Baker, 2008). Finally, we have also argued that language development improves children’s capacity to perform anaphoric reference to different given elements — either entities or propositions (Lambrecht, 1994) — in a conversation, which allows interlocutors to grammatically articulate them to their assertions.

In short, the faculty of language is responsible for giving rise to the different kinds of information that we can transmit or acquire through communication throughout our lives. Language does so by producing structures that are formed by a semantic ‘interior’ and a referential ‘edge’. These structures ground different forms of nominal reference, such as ‘a cat,’ ‘the cat,’ ‘this cat’ etc.,^16^ (Martin and Hinzen, 2014), as well as different forms of temporal reference, such as ‘he *refused* a job’. Assertions necessarily involve both temporal and nominal reference (the latter through the sentential arguments of the assertion), and their truth value seems to emerge as a ‘spandrel’ from the convergence of these ‘referentialities’ (together with other grammatical and prosodic features that mark the assertive character of the sentence). In taking the faculty of language as a merely symbolic system (as Enfield, 2009, and Tomasello and Herrmann, 2010, do), we cannot explain the ontology of the semantics involved — and consequently not its fundamental role in communicative learning either.

It is natural that as inquiry into language proceeds, our vision of what language is (ontology) changes along with our perspective on it (theory). A conventional formal definition of ‘language’ and ‘linguistic structure’ has widely influenced the language sciences. Although methodological abstractions such as those that are involved in the formalist paradigm can be well motivated at a time, they can also cease to be useful, as Chomsky (1965) in particular stressed. We have argued here that, instead of viewing language as an ‘encapsulated’ capacity with primarily formal properties, the faculty of language could be inherent to aspects of thought, meaning, and communication that are human-specific. This insight can also provide a new starting point for investigating language disorders and impact on their clinical definitions, which insofar as they involve the term ‘language’ are necessarily theory-dependent^17^.

All in all, language (as identified and described in the terms laid out in this article) could play a more essential role in cognitive development than often supposed, leading to the co-development of specific grammatical patterns and the different forms of human communication^18^. The range of this perspective could potentially be further supported through cognitive studies that explore the connection between referential linguistic structures and communicative and social abilities in neurotypical and neurodiverse populations in a comparative fashion, as well as neurophysiological and neuropsychological studies that aim to verify overlaps of our language circuitry with other cognitive capacities such as natural pedagogy.

## Conflict of Interest Statement

The authors declare that the research was conducted in the absence of any commercial or financial relationships that could be construed as a potential conflict of interest.

## Footnotes

1 In this paper we will mainly focus on declarative gestures, though we recognize the central role of ostensive signals not only for natural pedagogy, but for language use and development.

2 Whiten et al. (2009) criticized the idea that apes are exclusively emulators, suggesting that they are also able to *imitate* others’ strategies to achieve specific results. Be it as it may, for our present discussion it is enough to say that the transmission of traditions through communication is only observed in humans (Csibra and Gergely, 2006).

3 While we strongly agree with Iverson and Goldin-Meadow (2005) that ‘gesture and speech form a single integrated system,’ for these authors human gesture ‘paves the way’ for or ‘facilitates’ language development. By contrast, we suggest that infants’ declarative gestures are themselves the expression of emerging linguistic structures, structures that gradually become more complex throughout the development of the faculty of language. This perspective makes sense of the humanly unique features of declarative gestures such as their bipartite structure, the inherent intentionality (with a ‘t’) and intensionality (with an ‘s’) of the forms of reference involved (see further discussion at the end of this section), and their central role in the emergence of natural pedagogy.

4 Importantly, declarative gestures not only start out as part of our referential, linguistic system, but they crucially *remain* an inherent aspect of this system once it has developed fully. In particular, this kind of gesture is a fundamental ingredient in demonstrative reference with deictic expressions such as ‘this’ and ‘that’, which are universal (Diessel, 2006). Deictic reference has long been noted to be disturbed in people on the autism spectrum (Hobson et al., 2010), a disturbance that is, as we would predict, part of larger significant anomalies in the referential use of language in this population (Modyanova, 2009). Interestingly, deictic gestures do not seem to be impaired in children with SLI (Iverson and Braddock, 2011), and therefore we would expect them to have a better control of the grammar of nominal structure compared to children on the autism spectrum — although they do show problems with it as well, such as producing significantly more substitutions of definite articles than age-matched TD children (Polite et al., 2011; Chondrogianni and Marinis, 2015).

5 Our perspective in this sense is compatible with McNeill’s (2014) general view that some gestures and speech comprise a single, integrated multimodal system, while there are also early gestures not related to it. The latter, according to him, are quite different from gestures that are unified with speech in what he calls a ‘dual semiosis’ — i.e., when “gesture and speech become co-expressive rather than supplemental” (Levy and McNeill, 2015, p. 173).

6 For Povinelli et al. (2003), the reason behind children’s success in this experiment is their capacity for theory of mind, something that they claim to be absent in chimpanzees. We, on the other hand, suggest that their comprehension of declarative gestures is above all related to the referential mechanism of human language. The described study cannot exclude our position, which is supported by the evidence presented throughout this section. Independently of that, much evidence, reviewed in De Villiers (2007), suggests that full and explicit theory of mind is language-dependent. In this way, even if we attribute some form of theory of mind to one or another non-linguistic species, this does not mean that the members of this species think propositionally and have a capacity for intentional reference (see Fitch, 2010: P. 187–194).

7 Iverson and Thelen (1999) observe that speech and gesture are strongly synchronized in adults but not in children, even when gesture and vocalizations occur together. The authors then propose, based on neurophysiological and neuropsychological evidence, to account for the timing relationship between them throughout development as follows: “During the time when infants are just beginning to acquire many new words, speech requires concentration and effort, much like the early stages of any skill learning. As infants practice their new vocal skills, thresholds for hand–mouth activity decrease, and (…) (when) the level of activation generated by words is well beyond that required to reach threshold, it has the effect of capturing gesture and activating it simultaneously” (p. 35). In our view, this explanation accounts well for the fact that declarative gestures and ‘non-pronounced words’/meaningless vocalizations/words could still be connected to the same linguistic structure in infants’ mind, even when their gestural and oral production are not strongly synchronized yet.

8 Throughout this paper, we assume a crucial distinction between animal abstraction and human concepts, explicated in more detail in Hinzen and Sheehan (2013, ch. 2). Animals can form abstract perceptual stimulus classes, which order their experience in adaptive ways. This is a necessary but not a sufficient condition for human concepts. Concepts are abstractions that necessarily exist as the ‘interior’ of linguistic structures. These linguistic structures allow us to establish connections between the external and the internal world without the necessity of a perceptual mediation. In non-human animals, their perceptual input activates and ‘combines’ with their abstract knowledge, but human abstractions can be associated to linguistic ‘edges’ instead of percepts.

9 Full (explicit) theory of mind inherently involves an understanding of both intensionality and intentionality, since beliefs that we attribute to agents have both intentional contents (they are intentionally directed at objects), and these contents feature concepts that can give rise to intensionality effects (objects referred to do not have the properties that the concepts of them capture and vice versa). It is in line with the present viewpoint that there is extensive evidence for a developmental link between language, explicit theory of mind, and intensionality (Rakoczy et al., 2015), as well as language (specifically, the understanding of finite clausal complements around the fourth birthday) and explicit theory of mind (De Villiers, 2007; De Villiers and De Villiers, 2012). Further evidence for this link comes from children with autism spectrum conditions (Paynter and Peterson, 2010), and from overlaps in the neural correlates of theory of mind and the language comprehension network (Ferstl et al., 2008). Astington and Jenkins (1999) classical longitudinal study of 3-year old infants found that controlling for earlier theory of mind, earlier language abilities predicted later theory-of-mind test performance, while the reverse, controlling for earlier language, was not the case. On the other hand, theory of mind is arguably a composite function involving a number of different cognitive abilities, including face recognition (in seeing infants), empathy, tracking intentions and goals, and other abilities besides language.

10 Tomasello (2006, p. 520) suggests that “asking why only humans use language is like asking why only humans build skyscrapers (…) (and so) asking why apes do not have language may not be our most productive question. A much more productive question (…) (is) why apes do not even point”. But it follows from our account that these two questions are precisely related: the answer why apes do not point may lie on the fact that they do not have a faculty of language.

11 In formal terms, a predicate of the form ‘dog’ that is part of a pointing gesture at the proto-DP stage need not automatically be interpreted non-propositionally, after a translation into a formal language. That it corresponds to a proposition would mean that the child, effectively, is expressing the proposition that the object pointed to is a dog. In this case, there are propositions the moment that there are pointing gestures. In particular, where ‘dog’ is a noun, (_N_ dog), the property of being a dog obtained through abstraction would be x. dog(x). The formula [dog(x)]*^g[a/x]^* can then be defined as true in model M, iff the individual constant *a* is a dog in M under the variable assignment *g*. A child’s act of pointing can now be understood as an assignment in this sense, and the reinforced pointing gesture as conveying the proposition that the object pointed at is a dog. We don’t question that such a formal translation is possible. Our empirical claim is that, at the point of the proto-DP, a full model in which propositions can be *cognitively* evaluated as true or false is not yet available. We thank Hannes Rieser for conversations on this issue.

12 Prasada (2000) is not talking about statements with statistical prevalence like “all dogs are four legged” or “X% of dogs are four legged”. According to the author, knowledge about kinds allows us to “explain the existence of an essential property in an exemplar by citing the kind of thing it is” (Prasada, 2000, p. 66), as in the following example cited by the author on page 67:    Why does that have four legs? (pointing at a dog)    Because it is a dog.

13 We are not saying here that determiner phrases cannot specify temporal information lexically, in their ‘interior’ (the NP-part of a complex DP), which a simple example like ‘John’s smile at last night’s party’ would be enough to falsify. We are claiming that a complex DP like this one is crucially different from a sentence like ‘John smil*ed* at last night’s party’, which establishes temporal deixis grammatically. In the former expression, which unlike the latter cannot as such be true or false, the prepositional phrase ‘at last night’s party’ descriptively precisifies the assigned referent. In the latter, the verbal inflection does not have any descriptive function for the referentiality of the sentential argument (‘John’), but sticks a new referential ‘flagpole’ (a temporal one) to which the lexical concept ‘smile’ is attached. The result is reference to an event as opposed to an object, together with a temporal relation of this event to the time of the speech event.

14 Klein (1998) illustrates this point with the sentence ‘John was dead.’ Unless you believe in the possibility of resurrection, John is still dead and will continue being dead. Therefore, the finite element ‘was’ only indicates that he supposedly died before the time of utterance, not the end of the situation.

15 There is a dispute regarding whether children can also use pointing to ‘now-empty locations’ to indicate an object that is no longer present (see Liszkowski et al., 2007, for a defense of this claim and Southgate et al., 2007, for a criticism of it). Here this discussion is not fundamental because in both cases pointing has a deictic function (i.e., children use it in contingence to the immediate surrounding world, even if they are trying to denote a ‘now-absent object’). Be it as it may, we will adopt for explanatory reasons the claim made by Southgate et al. (2007) that children can only use pointing in reference to present or occlude objects.

16 Not forgetting, as we mentioned in the Section “Declarative Gestures: Language’s Illegitimate Child”, that the position occupied by the determiner phrase in the sentence structure can prevent referentiality. In this way, in the sentence ‘that guy is a thief,’ the determiner phrase ‘a thief’ works as a predicate, not picking out any referent.

17 This in particular concerns aspects of language impairment in Autism Spectrum Disorders, Specific Language Impairment, and Schizophrenia, on which we have commented elsewhere (Hinzen et al., 2015; Hinzen and Rosselló, 2015; for a synthetic statement see Hinzen and Sheehan, 2013, ch. 8). In all of these cases, language deviance may be an inherent aspect of core symptoms.

18 This would be in line with the ‘un-Cartesian’ linguistic project of Hinzen and Sheehan (2013), which, as a program of research, does not separate human-specific forms of thought, reference, and communication from the forms of grammatical complexity with which they co-occur in our species and from which it appears they cannot be separated.
